# On the Submersion of Swallows

**Published:** 1808-04

**Authors:** W. Johnson


### *On the Submersion of Swallows.*

[ Extracted from the New York Medical Repository. ]

THE substance of what is contained in the enclosed letter, was related by the writer, in an accidental conversation on the disappearance of Swallows. As he had preserved a memorandum of the facts, and the utmost reliance could be placed on the accuracy of the statement, I thought it of too much importance, in relation to a much agitated question in natural history, to be withheld from the public. More particularly as, from its coincidence in time, it may serve to confirm a similar fact, stated in the New York Medical Repository, vol. ii. p. 178, first edition, as observed by Mr. Peter Cole, in this city; the truth of which is questioned by an anonymous writer in the 3d vol. p. 241, of the same work, who regards the opinion of the submersion of swallows as exploded. Mr. Pollock has obligingly complied with my request to make the fact known, by sending me the enclosed, with liberty to insert it, with his name, in the New York Magazine. That the swallows could descend, in spite of their specific levity, levity, to the bottom of so deep and rapid a river as the Hudson, or remain there during the winter, is not, perhaps, to be supposed *. Yet the fact of their *submersion*, after the testimony of Mr. P. and Mr. S. men of undoubted veracity, cannot be questioned. Their continuance in a torpid state, and re-appearance, are different questions, which remain to be decided. The apparent impossibility of their existence under water, arising from their peculiar organization, should make us very doubtful, but not absolutely to reject the utter possibility of the fact. For "natural history," says Kalm, who, with the rest of the Swedish naturalists, defends the *hybernation* of swallows, in lakes, ponds, marshes, and caverns, "as all other histories, depends not always on the intrinsic degree of probability, but upon facts founded on the testimony of people of noted veracity." Reasonings and conjectures on the fact here stated, I leave to naturalists. It is to be hoped, that it may not be thought unworthy the notice of the learned, candid, and ingenious Dr. Barton, who has already bestowed so much attention on the subject.

W. JOHNSON.

"ON the afternoon of the 24th of August, 1798, I was sitting in my parlour which looks towards the North River, about fifty feet from the bank, in company with our mutual friend, Mr. Jacob Shebor. Our attention was attracted by numerous flights of birds, which appeared to come across the town from the eastward, and descend immediately into the river. So singular an appearance excited our particular observation. We went out and stood close to the bank, and then perceived that what we at first imagined to be black-birds, were actually swallows; and that, as soon as the various flocks had cleared the house, and got directly over the river, they plunged into the water and disappeared. This was not confined to the vicinity of the place where we stood, but was the case as far as the eye could reach, up and down the river, and continued, without cessation,

* The house of Mr. Pollock is situated near the margin of the Hudson, about two hundred yards from the battery. The river is about a mile and an half wide, and from seven to nine fathoms deep, and runs with a strong and rapid tide. Mr. P. does not recollect the species of swallow which then disappeared. The Barn Swallow (*Hirundo rustica,*) Chimney Swallow (*Hirundo pelasgia,*) the Sand or Bank Martin (*Hirundo riparia,*) and the Purple Martin (*Hirundo purpurea,*) all frequent and build their habitations in the city and its neighbourhood.

sation, for nearly two hours, when the closing of the evening prevented our further observation.

"Aware of the importance of affording any additional information on this long disputed question in the natural history of the swallow, I procured a telescope, and watched attentively many of the flocks from their first appearance until their immersion, continuing my eye fixed upon the spot long enough to be fully convinced that not one of the birds returned to the surface again. Indeed, one flock of about two hundred birds plunged into the water within thirty yards of us, and instantly disappeared, without the least appearance of opposition that might be expected to arise from their natural buoyancy, and, at the same time, the evening was so serene, and the river so unruffled, that no deception of our sight could possibly have occurred.

"When the birds first came in view, after crossing the town, their flight was easy and natural; but when they descended nearly to the water, they appeared much agitated and distressed, flying in a confused manner against each other, as if the love of life, common to all animals, impelled them to revolt against this law of nature imposed upon their species.

As some time elapsed since the above-mentioned facts occurred, I thought it proper, before I gave you Mr. Sebor's name, as having been a witness to them, to consult his recollection on the subject, and I have pleasure in assuring you he distinctly remembers every circumstance I have recited, and of which I have made a memorandum at the time.

"It may be worthy of remark, that as far as my observation went, the swallows totally disappeared on the 24th of August, 1798; for, during the remainder of that year, I did not see one." H. POLLOCK.

*New York, July* 18, 1800.

